# The Validity and Reliability of Motion Analysis in Patellar Tendon Reflex Assessment

**DOI:** 10.1371/journal.pone.0055702

**Published:** 2013-02-07

**Authors:** Lai Kuan Tham, Noor Azuan Abu Osman, Wan Abu Bakar Wan Abas, Kheng Seang Lim

**Affiliations:** 1 Department of Biomedical Engineering, Faculty of Engineering, University of Malaya, Kuala Lumpur, Malaysia; 2 Division of Neurology, Faculty of Medicine, University of Malaya, Kuala Lumpur, Malaysia; University of Iowa Carver College of Medicine, United States of America

## Abstract

**Background:**

The deep tendon reflex assessments that are essential to the accurate diagnosis of neurological or neuromuscular disorders are conducted subjectively in clinical neurology. Our aim was to assess deep tendon reflexes objectively with a new reflex quantification method.

**Methodology/Principal Findings:**

The present study used a motion analysis technique to collect quantitative measurements for both the input and output of normal patellar tendon reflex. Reflex responses were measured as knee angles. The patellar tendon reflexes of 100 healthy subjects were examined using 6 levels of tendon taps, where all the assessments were captured using motion capture system. A linear relationship was found between the experimental maximum tapping velocity and tapping angle (coefficient of determination = 0.989), which was consistent with the theoretical values. Tapping velocities were predictable according to tapping angles. The findings proved the reproducibility of tapping method in producing consistent input. The reflex amplitude was consistent between two randomly assigned groups, and linearly proportionate to the tapping velocity.

**Conclusions/Significance:**

The findings on reflex amplitude indicate that motion analysis is a valid and reliable method of assessing and measuring deep tendon reflexes.

## Introduction

The deep tendon reflex assessment is an essential element in neurologic examination to diagnose neurological or neuromuscular disorders [1]–[8]. Tendon reflex is useful clinically in evaluating the functional disturbance of either a normal or augmented reflex arc [5], [9].

Clinical evaluation on tendon reflex is qualitative and subjective, leading to great variation in the assessments [10]. According to a study on the effects of reflex stimulus to reflex responses by Stam and Tan (1987), reflex response could be affected by various factors, such as tapping force, direction and location of the impact. Thus, standardized tendon taps are required to reduce the variability in human reflex assessments [11], [12].

The acknowledgement that current methods of assessing tendon reflexes are subjective has lead to wide research into the ways in which reflex stimulus can be quantified. Significant effort has been placed into developing various kinds of instrumented device to produce accurate taps on human tendons. Studies to quantify reflex input generally involved the development of automated tapping devices [13], [14] and manual instrumented reflex hammers [3], [15]–[17]. The solenoid-type reflex hammer developed by Simons and Lamonte (1971) operated using an electronics control system exerted taps to the tendon automatically. Instrumented hammer involved those of Stam and van Crevel (1989), attaching an ordinary reflex hammer to a piezo-electric transducer, and of Mamizuka et al. (2007), who collected quantitative input data by placing a force sensor at the hammer tip. Surface electromyography (SEMG) is most commonly applied as the method to quantify reflex responses [3], [13]–[16]. Other methods include attaching force transducer [18] and accelerometer [17], [19] to the joint.

In order to deliver tendon taps at the exact location, the subject was often fixed at a specific position with restricted movements. This is different from the clinical method that does not restrict the joint movements [12], [13]. In addition, the instrumented tapping devices yielded greater reflex variability compared to the simple hand-held reflex hammer. There was also evidence suggesting that tendon taps produced by the instrumented hammer were unpleasant to the subjects [20]. Certain automated tapping devices [12] involved bulky experimental setups that would be hard to apply in clinical settings. The application of EMG to measure reflex responses is very convenient, but problems frequently arise in relation to the placement of the electrodes [21].

Motion capture systems are commonly used to analyze the joint kinematics and kinetics of human locomotion [22]. The authors of present study proposed a new alternative of assessing deep tendon reflexes using the technique of motion analysis [23]. The technique allows experiments to be conducted without altering clinical procedures or restricting movements of the subjects. The present study assessed the validity and reliability of this method in quantifying the input and output of normal patellar tendon reflexes.

## Materials and Methods

The study protocols have been approved by a review board at the Department of Biomedical Engineering of the University of Malaya, Malaysia. Written consent was obtained from all subjects before the experiments.

One hundred subjects consisting of 50 males and 50 females aged between 21 and 32 years were involved in the study. The mean age, height and body mass for male subjects were 24.8±2.1 years, 170.4±5.8 m and 65.1±9.9 kg respectively. Female subjects had a mean age of 24.7±2.4 years, mean height of 160.2±5.2 m and a mean body mass of 51.7±5.9 kg. All subjects were healthy with no history and recent record of neurological disorders. Subjects with neurological disorders that might exhibit abnormal reflex responses were excluded from the study. The Queen Square reflex hammer was used to examine patellar tendon reflexes in the study. Three reflective markers (14 mm diameter) were attached on the reflex hammer; one was positioned 5 cm below the tip, one at half the length of the handle and one on the rubber ring of the hammer head. Using a screw, a protractor was fixed to the tip of the reflex hammer in order to measure the tapping angle.

A total of 16 reflective markers (14 mm diameter) were attached to the lower body of the subjects following the Plug-in-Gait Marker Placement [24]. All tests on patellar tendon reflex were conducted by a trained physician with the intention of collecting reflex data in circumstances that mimicked clinical reflex examination. The motion capture software, Vicon Nexus 1.6 (Oxford Metrics, Oxford, UK) was used to capture the experiments at a sampling rate of 50 Hz.

The subject was seated upright on a high platform with both legs dangling. To avoid discomfort that could affect the natural reflex responses, the motions of the body were not limited. The mid-point of the patellar tendon between the lower border of the patella and the tibial tuberosity, which exhibited the greatest reflex response, was identified. The location was marked as the target spot for tapping. The reflex hammer fixed to the protractor was held parallel (0°) to the patellar tendon, where the hammer’s head was at the same level with the identified spot. The reflex hammer was raised to a specific angle and released, tapping on the patellar tendon. No external force was exerted on the reflex hammer. For each experiment, the patellar tendon reflex at both left and right sides was assessed using a tapping angle of 15°, 30°, 45°, 60°, 75° and 90°. The tendon was tapped 5 times at every tapping angle; each tap was delivered 10 to 15 seconds after the response stopped. The trial with the best response among the 5 taps was documented.

The trajectories of the markers attached to the reflex hammer were obtained from the experiments to calculate the velocity components *v_x_*, *v_y_* and *v_z_*. The average velocities of the hammer’s head at all captured frames were calculated using the formula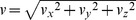. The experimental maximum velocity was compared to the velocities calculated using the theory of conservation of mechanical energy, as 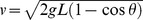 where the gravitational acceleration (*g*) = 9.81 m/s^2^, the handle length of the reflex hammer (*L* ) = 0.314 m and *θ* is the tapping angle in degree (°).

**Figure 1:**
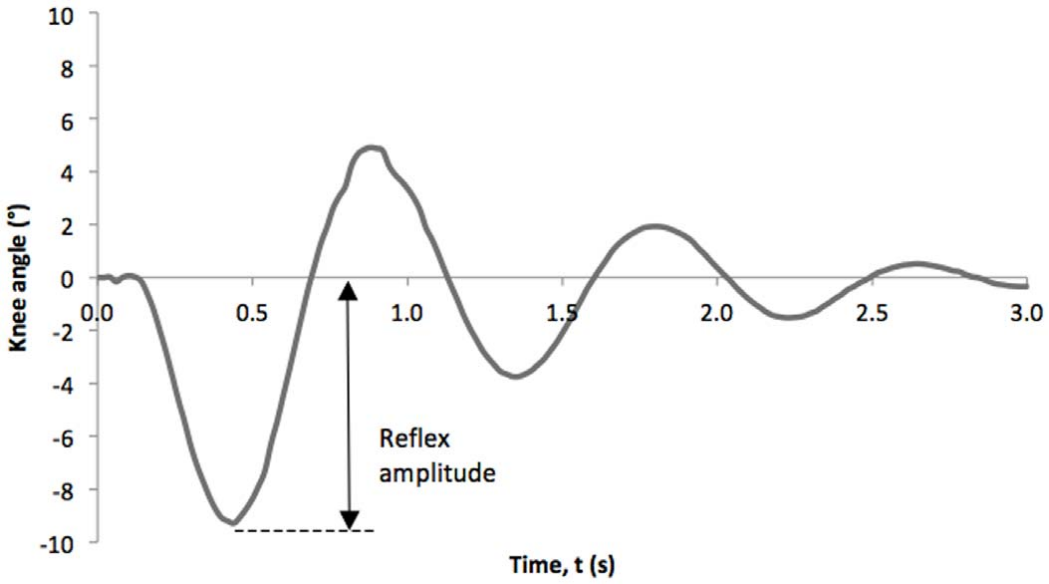
Representative knee angle of a patellar tendon reflex test. Time 0 is the time the reflex hammer struck the patellar tendon. Reflex amplitude is measured as the offset of knee angle from the original position.

An example of the knee angle tracing in a single trial is shown in Figure 1. Reflex amplitude, indicating the range of motion of knee in a reflex, is defined as the offset of knee angle from the resting position.

Statistical analyses of the data were carried out using SPSS for Windows (Version 20; IBM Corp., Armonk, NY). The reliability of the input method was tested using (1) one sample *t*-test to determine the significance of differences between the experimental and calculated maximum velocity, and (2) linear regression analysis to determine how tapping angle predicts the experimental maximum tapping velocity. Absence of significant differences between the experimental and calculated maximum velocity, and a coefficient of determination (*r* ^2^) close to one was deemed to support the reliability and predictability of the tapping input in the study.

The reliability of the output assessment was tested by comparing the reflex amplitudes of two random groups using student *t*-test. The absence of significant differences in reflex amplitudes between the two groups was deemed to support the reliability of the output assessment.

The validity of this method was tested using a linear regression and correlation analyses between the tapping velocity and reflex amplitude. Using a hypothesis that a larger tapping velocity produces a stronger reflex response, as measured by reflex amplitude [12], a strong correlation with coefficient of more than 0.5 was deemed to support the sensitivity of this method in measuring the reflex response. The paired *t*-test was used to determine the statistical differences between reflex amplitudes of different tapping angles. Statistical significance of *P*<0.05 was determined for all comparisons.

## Results

**Figure 2:**
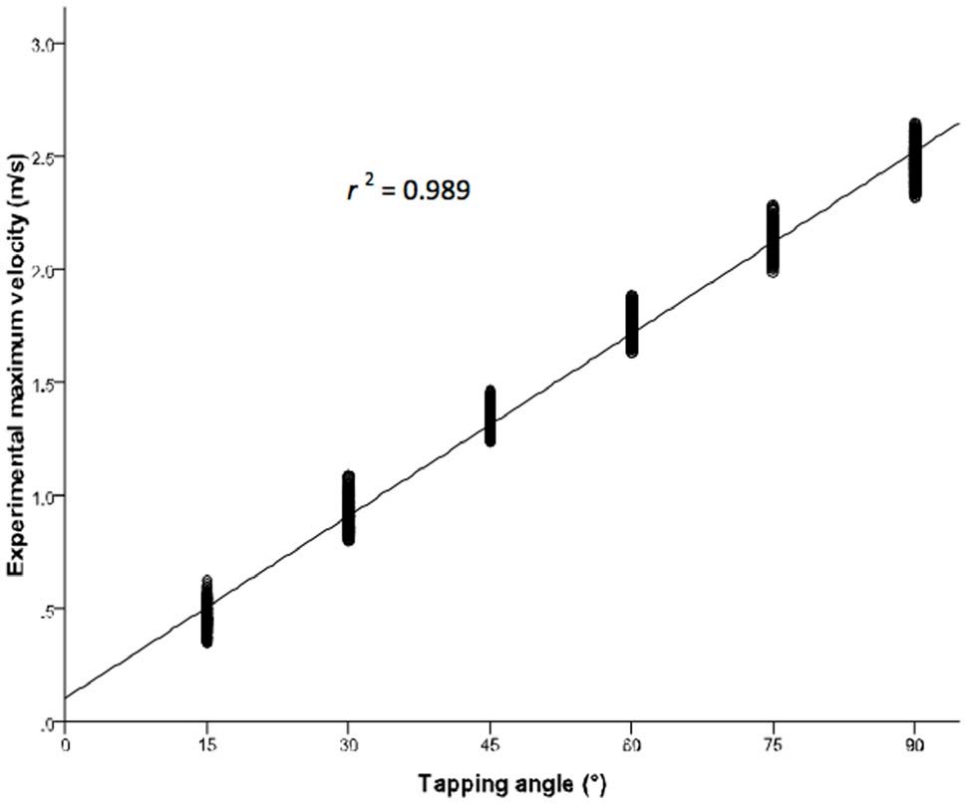
Representative relationship between the tapping angle and the experimental maximum tapping velocity.

The experimental and theoretical values of maximum tapping velocity for the six different tapping angles are shown in Table 1. The one sample *t*-test found no significant differences between experimental and theoretical maximum velocity at all tapping angles. A linear relationship between the tapping angle and the experimental maximum tapping velocity is demonstrated in Figure 2, with coefficient of determination close to 1 (*r* ^2^ value of 0.989).

**Table 1:** Comparison of mean values of experimental and theoretical maximum tapping velocities (±SD).

Tapping angle (°)	Maximum velocity, *v_max_* (m/s)		*P* value^b^
	Experimental (*n = *200)	Theoretical	
15	0.466±0.059^a^	0.458	0.059
30	0.918±0.066^a^	0.909	0.066
45	1.345±0.051^a^	1.343	0.601
60	1.758±0.064^a^	1.755	0.450
75	2.130±0.061^a^	2.137	0.100
90	2.472±0.077^a^	2.482	0.061

a *P*<0.001 when compared to the maximum velocity of other tapping angles.

b Comparison between experimental and theoretical maximum velocities.

**Figure 3:**
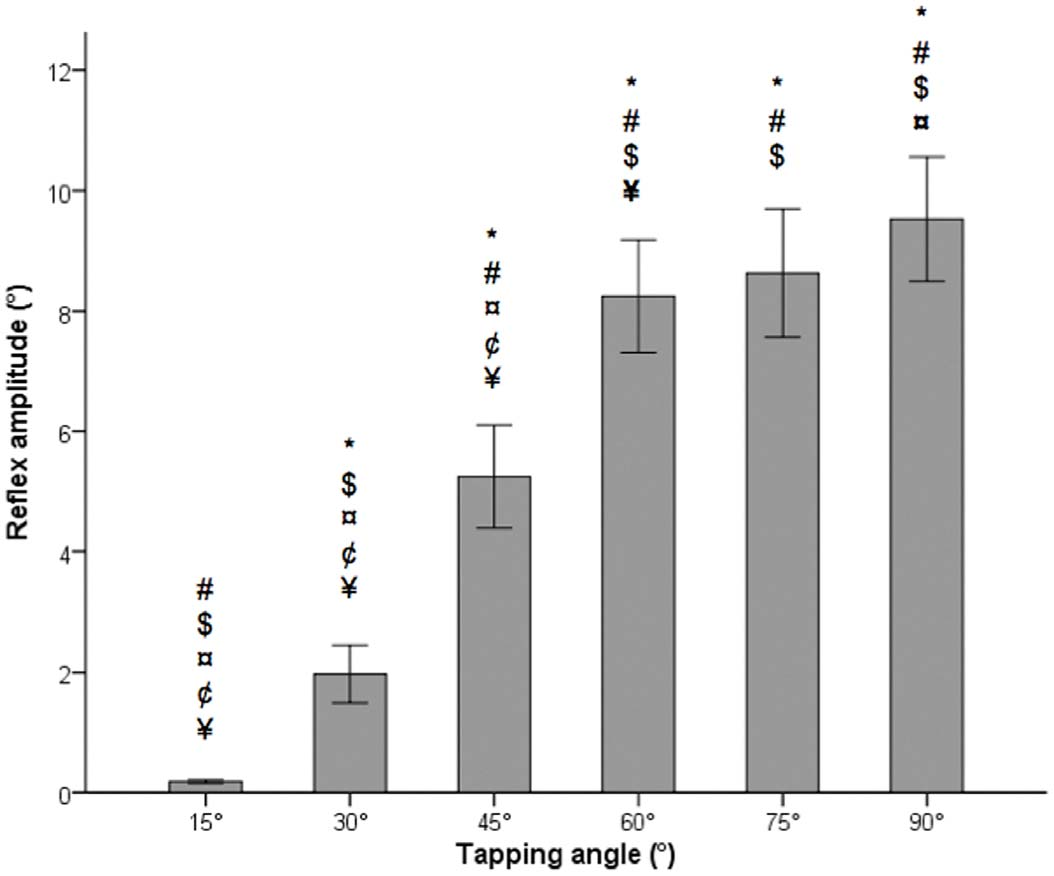
Mean values of patellar tendon reflex amplitudes according to tapping angle (± standard error). The reflex amplitude increased gradually with the increment of tapping angle. ^*^ denotes statistical significance difference compared to tapping angle of 15°. ^#^ denotes statistical significance difference compared to tapping angle of 30°. ^$^ denotes statistical significance difference compared to tapping angle of 45°. ^¤^ denotes statistical significance difference compared to tapping angle of 60°. ^¢^ denotes statistical significance difference compared to tapping angle of 75°. ^¥^ denotes statistical significance difference compared to tapping angle of 90°. Symbols (^¤^, ^¥^) in bold indicates statistical significance difference at *P*<0.01, symbols not in bold indicates statistical significance difference at *P*<0.001.

**Figure 4:**
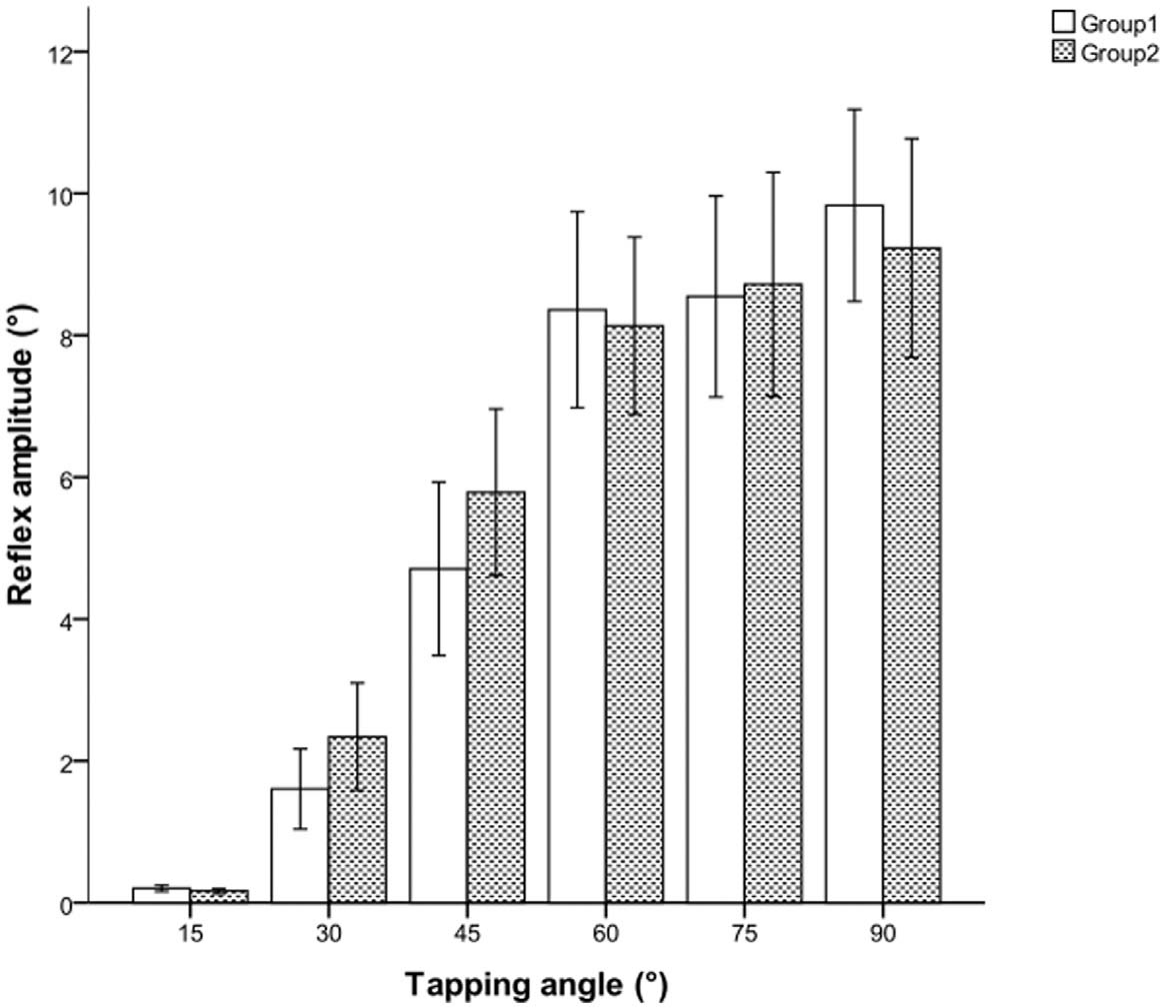
Reflex amplitude of random groups in mean (± standard error) according to tapping angle.

The quantified values of the normal patellar tendon reflex amplitude according to tapping angle are presented in Figure 3. In general, reflex amplitude increased as the tapping angle increased. Reflex amplitudes were significantly lower for small tapping angles of 15°, 30° and 45°, representing low reflex inputs. The increment of reflex amplitude reduced as the tapping angles increased, the differences in reflex amplitude were not significant for the comparison pairs between tapping angles 60° and 75°, and 75° and 90°. The comparisons of reflex amplitude between two random groups (50 subjects in each group) found no significant difference for all tapping angles (Figure 4).

The reflex amplitude was linearly related to the tapping input with an equation of *y* = 4.9*x*–1.8, in which *y* is the reflex amplitude (°) and *x* is the maximal tapping velocity (m/s). The correlation between the tapping input and reflex amplitude was strong (Pearson’s correlation coefficient was 0.501, *P* value <0.001).

## Discussion

The present study described the quantification method of patellar tendon reflex in healthy adults using motion analysis. The reflex input was also characterized according to tapping angles, in which applying predetermined inputs will ensure the consistency of reflex stimulus [12].

The reliability of the tapping method was supported by (1) a linear relationship between the tapping angle and the experimental maximum tapping velocity, (2) small variances in experimental maximum velocity elicited in any tapping angle, and (3) non-significant differences between experimental and theoretical maximum velocities. These findings proved the reproducibility of the proposed tapping method. Consistent and reproducible tendon taps are critical to improve the reliability and sensitivity of reflex assessment [11]. Moreover, the non-significant differences in the comparisons of reflex amplitude between two random groups indicate that the output obtained is consistent.

Based on the hypothesis that a larger tapping angle generates greater stimulus on the muscle receptors that would ease the development of action potentials [12], the linear relationship between tapping input and output proved the validity of this method in measuring reflex output. The significant differences in reflex amplitude between different tapping angles (Figure 3) showed that this method is sensitive to the detection of output variation according to tapping input.

In the current study, the reflex amplitudes were quantified according to tapping angle. Tendon tap at small tapping angles, such as 15°, did not exert sufficient stimulus to the tendon and produced only slight reflex responses. This is comparable to the results of a previous study [12]. Significant differences in reflex amplitude was noted between tapping angles of 30° and 45°, and 45° and 60°, suggesting that 45° or 60° is the best tapping angle to elicit an adequate response in clinical practice. Larger tapping angles produced greater output, however, the increment of output with a tapping angle larger than 60° was minimal and not significant. This provides a basis for a tapping angle of 60° to be used in daily clinical practice to obtain optimal tendon reflex response. The patellar tendons were tapped sequentially from 15° to 90°, not in a random order, might contribute to the plateau of the reflex response at the higher angles. Random reflex input is therefore suggested to improve the accuracy of the results in the future.

The proposed method provides quantitatively valid and reliable measurements of tendon reflex. Inter-rater variability in the clinical examination of the patellar tendon reflex has been resulted from the variability in the way clinicians elicit the reflex and assess the response [25]. In this study, the inter-rater variability was minimized using a standard elicitation method and the aid of an automatic assessment tool. This technique is also capable to characterize tendon reflex into more kinematic and kinetic parameters than some previously developed reflex quantification systems which involved sensors or EMG that provided few measures of tendon reflex [3], [15], [17]. This method is sensitive to detect a small reflex response of less than 5°, which could hardly be observed in clinical assessment. The accuracy, repeatability and sensitivity of current method showed its feasibility to quantify deep tendon reflex in a clinical experiment.

Though the setup using markers and cameras limits its usefulness in daily clinical practice, it can be setup easily in a clinic or ward to ease patient recruitment in a clinical study. The proposed method is simple and does not necessitate complicated setups compared to systems that are tethered or fixed to static or bulky structures [12], [13], [19]. Major fixation such as limb casting or brace coupled with sensor [12], [17] is not required on the subject. The present method is also not affected by power line interference and electrode artifacts compared to the EMG systems [17]. In addition, this method, using an ordinary reflex hammer instead of an instrumented tapping device that incorporates force sensor, allows a simulation closer to daily clinical practice.

Motion analysis is a valid and reliable quantitative method to assess deep tendon reflex response. This technique, which is simple, non-invasive and non-tethered, permits free movements to the subject and therefore helps to reduce the degree of discomfort and anxiousness associated with the tests. Meanwhile, the examiner can perform reflex tests without being restricted by complicated experimental setups. The proposed method is capable to quantify other deep tendon reflexes in a manner that produces an objective and reliable assessment.

### Conclusions

Motion analysis is a valid and reliable method of assessing patellar tendon reflex objectively. This method can be replicated in the assessment of other deep tendon reflexes and has a great potential to improve the accuracy and reliability of clinical reflex assessment.

Tapping input was found to have significant effect on reflex amplitude. The present study suggests that 45° or 60° is the optimum tapping angle to elicit adequate reflex response.
